# Surfaces from the Visual Past: Recovering High**-**Resolution Terrain Data from Historic Aerial Imagery for Multitemporal Landscape Analysis

**DOI:** 10.1007/s10816-017-9348-9

**Published:** 2017-08-21

**Authors:** Christopher Sevara, Geert Verhoeven, Michael Doneus, Erich Draganits

**Affiliations:** 1 Department of Prehistoric and Historical Archaeology, University of Vienna, Franz-Klein-Gasse 1/III, Vienna, Austria; 2 LBI for Archaeological Prospection and Virtual Archaeology, Ludwig Boltzmann Gesellschaft GmbH, Franz-Klein-Gasse 1, Vienna, Austria; 3 Department of Geodynamics and Sedimentology, University of Vienna, Althanstrasse 14, Vienna, Austria

**Keywords:** Landscape archaeology, Image-based modelling, Geomorphic change detection, Historical DEM, Topographic bias, Sicily

## Abstract

Historic aerial images are invaluable sources of aid to archaeological research. Often collected with large-format photogrammetric quality cameras, these images are potential archives of multidimensional data that can be used to recover information about historic landscapes that have been lost to modern development. However, a lack of camera information for many historic images coupled with physical degradation of their media has often made it difficult to compute geometrically rigorous 3D content from such imagery. While advances in photogrammetry and computer vision over the last two decades have made possible the extraction of accurate and detailed 3D topographical data from high-quality digital images emanating from uncalibrated or unknown cameras, the target source material for these algorithms is normally digital content and thus not negatively affected by the passage of time. In this paper, we present refinements to a computer vision-based workflow for the extraction of 3D data from historic aerial imagery, using readily available software, specific image preprocessing techniques and in-field measurement observations to mitigate some shortcomings of archival imagery and improve extraction of historical digital elevation models (hDEMs) for use in landscape archaeological research. We apply the developed method to a series of historic image sets and modern topographic data covering a period of over 70 years in western Sicily (Italy) and evaluate the outcome. The resulting series of hDEMs form a temporal data stack which is compared with modern high-resolution terrain data using a geomorphic change detection approach, providing a quantification of landscape change through time in extent and depth, and the impact of this change on archaeological resources.

## Introduction

Historical aerial imagery has been put to many uses in research contexts that are secondary to its original purposes. Their original uses notwithstanding, historic aerial and spaceborne images have become essential resources for archaeological research, including archaeological landscape reconstruction, site location and monitoring (*e.g.* Comer and Harrower 2013; Cowley *et al.* 2010; Doneus 1997; Doneus 2000; Hanson and Oltean 2013; Kennedy 1996; Moscatelli 1987; Stichelbaut *et al.* 2009; Strandberg 1967). This is particularly true in Italy, where blocks of vertical photography acquired for nonarchaeological purposes have, until recently, been crucial components for archaeological research (Guaitoli 2003). Millions upon millions of images, many collected for photogrammetric purposes, exist in archives around the world (Cowley and Stichelbaut 2012; Cowley *et al.* 2013), archives which can therefore be seen not only as repositories for historic imagery but also as potential repositories of 3D historic terrains and built environments, many of which have long since changed, or vanished altogether. Rapid topographic changes related to agricultural production, resource extraction and population growth, as well as environmental issues related to climate change, have led to significant modification in the land surface in many parts of the world since the middle of the last century. For instance, it is estimated that the land take, which is the land surface taken by infrastructure itself and related facilities, in the European Community alone was around 1080 km^2^/year between 2000 and 2006 (EEA 2017). Many of the resources in these archives capture the past at key points prior to the beginning of such change. This is apparent in the western Sicilian context, an area with a long history of human occupation and one that has experienced rapid and intense changes in land use since the middle of the last century. Fortunately, these changes have been relatively well documented through repeated, systematic vertical aerial coverage campaigns carried out by a number of agencies, including the Italian Military Geographic Institute (IGM). Available IGM imagery of the area dates back to 1941, allowing us a 75-year window to evaluate changes in land use in the region.

Usually, vertical photographs are captured in blocks of overlapping strips covering larger areas. Within each strip, individual images usually overlap by at least 60%. As a result, each part of the documented area is covered from at least two stereo-photographs and can be analyzed in 3D. Vertical image archives therefore offer innumerable 3D-windows into the historic environment, although image use can be somewhat limited in geospatial contexts due to physical degradation of source media and a lack of available metadata necessary for processing in strict photogrammetric workflows (Redecker 2008). However, computer vision-based techniques that allow for the extraction of 3D data from imagery without prior information regarding camera parameters can now allow us to step through those windows and investigate the historic environment in multiple dimensions. These techniques provide the possibility to reconstruct historic digital elevation models (hDEMs) from archival imagery whose surfaces correspond to the date the imagery was acquired (Ishiguro *et al.* 2016; Sevara 2013, 402; Verhoeven *et al.* 2013; Verhoeven and Vermeulen 2016), even when camera information is missing or incomplete. Here, we consider ‘DEM’ to be a generic term that encompasses all digital encodings of elevation data. Stacking a series of hDEMs together results in a four-dimensional spatiotemporal ‘terrain data cube’ which can be used for diverse analytical tasks such as site degradation monitoring, historic landscape change analysis and reconstruction of the historic built environment. In particular, such datasets offer us the opportunity to evaluate the bias that the process of modern, large-scale physical modification of the landscape may impart to our interpretation of preceding human activity. In that way, they allow us to come some way toward understanding the impact of such bias on our interpretation of the archaeological record (Cowley 2013, 76; van Leusen 2002, chapter 11, page 17). Furthermore, observing the physical process of landscape change by modelling the differences in terrain from regular intervals provides insight into the dynamic and nonlinear nature of landscape and site formation processes.

In order to achieve this, we use a refined workflow for the processing of historic frame imagery into 3D geospatial content, using well-known image-based modelling (IBM) and geospatial data processing applications to generate a series of hDEMs for landscape analysis in western Sicily (Italy). This process is detailed and exemplified using a series of vertical photographs collected by the IGM over the Mazaro river corridor in western Sicily between 1941 and 1992. The resulting refined hDEMs are subsequently coregistered to an elevation model derived from Airborne Laser Scanning (ALS) data and stacked into a terrain data cube to be used to analyze terrain change through time and to estimate the effects on the visibility and condition of archaeological resources in the region. This approach provides an efficient and accurate way to differentiate areas of significant topographic gain and loss from areas that have remained relatively unchanged, information that can help us to understand why we see what remains of the archaeological record and to manage what is left.

## Historic Imagery: Processing, Concerns and Archaeological Context

### Background: Terrain from Imagery

Photogrammetric restitution of terrain information from historic imagery is a relatively well-known process that has been applied in numerous studies in which morphometric estimation of landscape change plays an important role. Recent archaeological examples include the use of historic elevation models for purposes such as multitemporal landscape analysis (Nocerino *et al.* 2012; Pérez Álvarez *et al.* 2013; Orengo *et al.* 2015) and to monitor the condition of specific archaeological sites (Papworth *et al.* 2016; Risbøl *et al.* 2014; Sauerbier *et al.* 2004). However, these applications generally tend to be based on rigorous photogrammetric workflows, which require known or reconstructable camera calibration parameters in order to be successfully applied. On the other hand, computer vision-based IBM workflows that utilize structure from motion (SfM) and dense image matching (DIM) algorithms such as the ones employed in this study can successfully reconstruct historic terrain data and generate orthoimagery from imagery which is heavily degraded or lacks calibration information necessary for processing in high-end photogrammetric packages (Ishiguro *et al.* 2016, 65; Sevara 2013, 2016; Verhoeven *et al.* 2012; Verhoeven *et al.* 2013; Verhoeven and Vermeulen 2016). Furthermore, this process is highly automated and relatively low-cost. Output quality of the resulting hDEM depends upon a number of factors, including original image quality and characteristics (Stylianidis *et al.* 2016, 260), image overlap, preprocessing, condition of the source material, the manner in which it was digitized and the quality of reference data used for georeferencing. Successful recovery of 3D data using this process brings a new dimension to the vast aerial and spaceborne imagery resources available in archives around the world, providing the potential to recover information about lost landscapes and by extension the historic character of the environment.

### From Analogue to Digital: Considerations and Best Practices

The conversion of analogue historical aerial imagery to digital geoinformation often involves considerations and issues unique to the medium. Unlike their modern digitally acquired counterparts, analogue images must be digitized from their film or paper sources before being converted into useable geospatial content. Up to and even during the process of digitization, analogue media are subject to various physical processes that can induce in them nonuniform radiometric and geometric degradation (Redecker 2008; Redweik *et al.* 2010). Additionally, wartime reconnaissance imagery may have been acquired under less than optimal circumstances, including those of poor lighting condition and/or visibility, semi-oblique camera positioning, variable altitude, extreme temperature change and other unstable flight conditions (Kingslake 1947, 6; Redecker 2008, 6). Choice of focal length coupled with lens aberrations and limitations to the resolving power of older lenses and film emulsions further restrict the clarity and visibility of objects captured on reconnaissance flights (Kingslake 1947, 7). Subsequent conditions for storage and transfer of images may have also been less than optimal. Finally, prints and film scans may originate from second or later generation transfers of the original media. All of these factors can impact the likelihood and ensuing quality of 3D data recovery.

When utilizing such resources in IBM contexts for the purposes of 3D reconstruction, these issues can have various negative effects on the processing outcome. Nonuniform geometric distortion caused by age-related degradation can cause localized errors, while the use of even high-end nonphotogrammetric scanners can cause nonuniform distortion of media during the digitization phase, including warping and stretching effects which can significantly affect terrain generation and which are also difficult to compensate for due to their very nature. Although calibration profiles that minimize the effect of geometric distortions caused during the scanning process can be built for nonphotogrammetric scanners (*e.g.* Nocerino *et al.* 2012), studies still show that nonphotogrammetric scanners which have been approximately calibrated are not able to produce digital imagery which is suitable for the most rigorous of photogrammetric tasks (Baltsavias and Waegli 1996, 19; Mitrovic *et al.* 2004). Furthermore, the time-consuming nature of manually applying calibration profiles to images scanned using nonphotogrammetric scanners may make such approaches less than ideal for large image collections (Mitrovic *et al.* 2004, 58). Therefore, scanning of imagery using photogrammetric grade devices should be considered essential for both present use and future preservation of scanned image content. Even then, a preprocessing workflow for image optimization will need to be applied prior to using them as a basis for terrain generation.

## Case Study Area and Datasets

### Western Sicily: Archaeological and Photographic Perspectives

Western Sicily has seen a long history of archaeological study that, in its infancy, focused mainly on large coastal colonial and ‘Hellenized’ indigenous sites. Although the interior region of the central western zone has received much more attention as discussions of various aspects of indigenous societies and colonial aspirations during the Bronze and Iron Ages have matured (*e.g.* Kolb 2007; Kolb and Tusa 2001; Leighton 1999, 2005; Morris *et al.* 2004; Morris and Tusa 2004, Mühlenbock 2008; Nicoletti and Tusa 2012a, 2012b; Spatafora 2003; Tusa 1972; Tusa 1999; and see De Angelis 2016 for a recent summary), human activity in the transition zone between coast and interior remains less well understood. Although areas in the wider region of western Sicily have been the subject of more systematic field survey in recent years (*e.g.* Blake and Schon 2010; and see Spanò Giammellaro *et al.* 2008 for a summary of survey projects in the region), the area around the Mazaro river has received comparatively little attention. The only recorded intensive surveys that fall within our project area were carried out on the north side of the Mazaro (Fentress *et al.* 1986; Mosca 2016). In the early 1980s, systematic survey revealed a large number of potential habitation sites and associated material from the fourth through first centuries BC (Fentress *et al.* 1986). This early work, which should be noted, called for the study of aerial imagery and use of geophysical prospection for further investigation in the area (Fentress *et al.* 1986, 87). Other work that has been done in the area, while of high quality, generally remains focused on particular ‘key’ sites and site/period concerns (*e.g.* Calafato *et al.* 2001; Mannino 1971) while the surrounding landscape has received comparatively little systematic investigation.

**Fig. 1:**
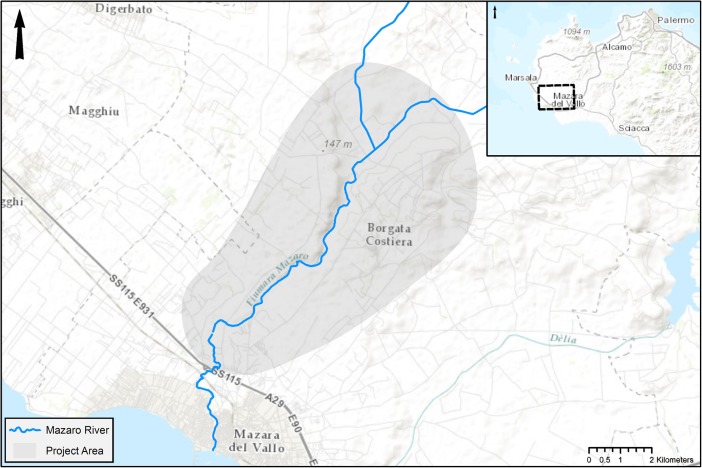
Location of project area, northeast of Mazara del Vallo, Sicily, Italy. Map background source: ESRI

The Prospecting Boundaries project (mazaro.univie.ac.at) seeks to change the focus of archaeological research in the region through systematic, noninvasive study of the landscape around the Mazaro river corridor. To do so, the project incorporates integrated archaeological prospection techniques to investigate the present remains of past activity in the landscape from both synchronic and diachronic perspectives. Centred on the Mazaro river, the project encompasses an area of roughly 70 km^2^, stretching inland from the river mouth at modern-day Mazara del Vallo (Fig. 1). The project is concerned with human activity during all periods in the region, working backward from the present to document and deconstruct modern and historical land use in order to try to connect the relict fragments of prior human activity to continuity and change in the wider landscape.

In our study, archival aerial imagery serves as a visual link between changing land use practices and their impact on our interpretation of the archaeological record in our project area. While the ‘direct’ appearance of archaeological sites (*e.g.* as relict earthworks or through the proxy of soil/crop discontinuities) in historic vertical imagery has often been equated with serendipitous discovery (Brugioni 1989, Fowler 2004), the appearance of historical land use and land cover could be seen as a more reliable manifestation in the historical visual record, this being a prime reason for its original acquisition. Intensive resource extraction, illicit waste disposal, unchecked development and large area agricultural transformation have had a significant effect on the region of western Sicily over the past 60 years, with a presumably significant corresponding effect on the archaeological record. To what extent this recent intensification of land use has had an effect on the visibility and preservation of archaeological resources is still relatively unquantified and moreover difficult to estimate in the present due to the presumed totality or near-totality of removal of archaeological resources in affected areas. However, due to Sicily’s strategic significance in World War II and subsequent intensive mapping campaigns by the IGM, an extensive amount of high-coverage historical vertical aerial imagery exists for the region from as early as 1941 (CRicd Regione Siciliana 2010). In addition to IGM coverage, wartime reconnaissance imagery of the region can be found at the National Collection for Aerial Photography (NCAP) archives in Edinburgh and the archives of the Aerofototeca Nazionale in Rome (Castrianni and Ceraudo 2009, 172; Ceraudo and Sheperd 2010; Cowley *et al.* 2010, 2013). Therefore, this transition zone, rich with archaeological resources, heavily affected by recent large-scale postdepositional processes, and historically well-photographed, provides an ideal location to explore the validity of historic terrain reconstruction for archaeological analyses.

### Historic Imagery

After initial research, images available from the IGM proved to be the best fit for our purposes in terms of overall image quality, coverage and time period. Historic images from four discrete points in time were acquired, providing a snapshot of landscape development at roughly 20-year intervals between the years of 1941 and 1992 (Table 1). The 1941 imagery was captured on glass plate negatives, using a Santoni glass plate multicamera, presumably a Model II, based on the size of the negatives (IGMI 2017; Nyssen *et al.* 2016, 173), and consisted solely of images emanating from the central (vertical) mapping camera. The other images were captured using Fairchild, Zeiss and Wild mapping cameras. All imagery was scanned at the IGM archives in Florence at a spatial resolution of 2500 samples per inch (spi), using photogrammetric scanning equipment to minimize introduction of any further geometric distortion that could affect the modelling process (Sevara 2016). Most image parameters, such as flight height, acquisition scale and side overlap, vary from flight to flight. Condition of the imagery also varies, with older images generally showing more signs of age-related degradation, including some areas of warping, scratches, radiometric decay and dust marks. Some camera parameters, including calibrated focal length, were available for all datasets with the exception of the 1941 imagery, for which calibration information was not available. All of these variables need to be taken into account when comparing results of various datasets produced from the IBM process, as the individual outcomes will be uniquely affected by some of these parameters and constraints.

**Table 1:** Parameters of imagery and ALS datasets used in this study

Dataset	Acquisition date	Scanning resolution	Source material	Scale	Focal length
257-4-106-109	22 Apr 1941	2500 spi	Glass plate negative	1:18,000 (est)	175 mm
257-28A-10998-11000	4 Jul 1955	2500 spi	Negative film	1:30,000	151.92 mm
257-28B-12102-12104	13 Jul 1955	2500 spi	Negative film	1:30,000	151.92 mm
257-IX-752-754	9 May 1975	2500 spi	Negative film	1:15,000	152.55 mm
257-VIII-770-774	9 May 1975	2500 spi	Negative film	1:15,000	152.55 mm
257-VII-788-791	9 May 1975	2500 spi	Negative film	1:15,000	152.55 mm
257-23-120-121	24 Jun 1992	2500 spi	Negative film	1:36,000	153.22 mm
257-24-1068-1072	24 Jun 1992	2500 spi	Negative film	1:36,000	153.22 mm
2016 RGB Image Capture	21 Feb 2016	39 MP	Digital capture	8 cm (GSD)	50 mm
2016 ALS Survey	21 Feb 2016	16 points/m^2^	Digital capture	25 cm (GSD)	60° (scan angle)
Dataset	Flight height	Image dimensions	No. of images	Overlap % (forward/side)	
257-4-106-109	3200 m	10 × 15 cm	4	70/n.a (variable)	
257-28A-10998-11000	6000 m	23 × 23 cm	3	60/n.a. (15–30)	
257-28B-12102-12104	6000 m	23 × 23 cm	3	60/n.a. (15–30)	
257-IX-752-754	2500 m	23 × 23 cm	3	60/20	
257-VIII-770-774	2500 m	23 × 23 cm	5	60/20	
257-VII-788-791	2500 m	23 × 23 cm	4	60/20	
257-23-120-121	6070 m	23 × 23 cm	2	60/30	
257-24-1068-1072	6070 m	23 × 23 cm	5	60/30	
2016 RGB Image Capture	511 m	7216 × 5412 px	316	60/30	
2016 ALS Survey	511 m	n.a.	n.a.	20/2 cross strips	

*spi* samples per inch, *est* estimated, *MP* megapixels, *GSD* ground sampling distance, *px* pixels, *n.a.* not applicable

### ALS Data Capture and Reference Datasets

Full-waveform (FWF) ALS data were acquired specifically for the project, along with simultaneously acquired RGB imagery. Airborne Technologies GmbH, a commercial provider of ALS surveys with experience in data acquisition for archaeological research, conducted the survey on the morning of February 21st 2016 using a Riegl LMS-Q680i scanner (Riegl 2012). Laser data and corresponding orthoimagery were collected in 26 longitudinal strips with an overlap of 20%, and two cross strips, resulting in a total average unfiltered point density of 16 points per square meter (Table 1). Due to this high point density, meaningful raster data products can be derived at a minimal cell size of 25 cm. Initial postprocessing, including strip adjustment and calculation of the 3D point cloud, was carried out in Terrascan (Terrasolid 2017). Subsequent generation of a digital surface model (DSM) with a raster cell size of 25 cm was carried out using the OPALS software package, using the last echo of the laser pulse (Mandlburger *et al.* 2009; OPALS 2017). The point cloud and last echo DSM generated from the ALS data capture were used for subsequent coregistration and comparison of historic models. Simultaneously acquired RGB imagery, at a ground sampling distance (GSD) of 8 cm, was also used for visual reference.

### Ground Points and Validation Dataset

A total of 54 ground points were collected across the project area in October of 2016, using a Leica Zeno 20 network real-time kinematic (RTK) enabled global navigation satellite system (GNSS) sensor connected to the Italian Positioning Service (ItalPoS) for correctional data (ItalPoS 2017). These ground points were used for two separate purposes: georeferencing of historic data (ground control points (GCPs)) and independent positional validation of the resulting models (checkpoints). Checkpoints collected for independent positional validation were not used as GCPs. Coordinates were collected in latitude/longitude format using the ETRF2000 reference frame and then converted to UTM. As ground control must naturally be acquired subsequent to imagery capture when using historic images, appropriate natural points were chosen for model georeferencing (Stylianidis *et al.* 2016, 268). These included corners of structures and other temporally static objects chosen *via* inspection of historic and modern imagery for identification of features in the historic imagery that could still be present in the modern landscape. Unfortunately, this means that nonoptimal targets, such as building corners, sometimes had to be chosen as they were the only relatively static objects in the landscape identifiable in both historic and modern contexts. Furthermore, all targets were not visible in all datasets. In cases where building edges were occluded, points were collected at opposing corners of buildings in order to ensure visibility.

In addition to their use in defining a coordinate reference system for the computed 3D surface model (georeferencing), a number of well-distributed GCPs are necessary constraints for the SfM bundle adjustment in order to avoid instability of the bundle solution (Remondino *et al.* 2012) and mitigate drift in both the recovered camera and the tie point locations (Snavely *et al.* 2006). When it is not possible to collect in-field measurements, control points can be acquired using a corresponding digital elevation model (DEM); however, the accuracy of the points and resulting 3D surface model will be limited by factors such as interpolation error and the spatial resolution of the source elevation model.

## Workflow: Conversion of Historic Imagery to Geocontent

As discussed in the section “From analogue to digital: considerations and best practices”, the nature of historic and archival imagery necessitates some unique considerations when converting it from analogue imagery to digital geocontent. Many parameters need to be accounted for that are not necessarily significant factors when recovering 3D information from modern digital imagery. Ideally, imagery should be sharp, well exposed and have a high degree of overlap between frames. However, historic aerial image sets do not always necessarily meet these prerequisites. In IBM workflows, the key challenge is the exact geometry and extent of the image network. The latter impacts both accuracy of the extracted dense surface and its completeness (Nocerino *et al.* 2014), because dense reconstructions based on multiview stereo need a suitable selection of stereo models. At a minimum, a feature should appear in two images for it to be reconstructed in 3D, although a feature that appears in more images can lead to an improved matching while this redundancy also benefits the point precision in object space (Wenzel *et al.* 2013). In the case of vertical imagery, a greater number of flight lines and/or more forward/side overlap as well as the incorporation of convergent imagery will thus always contribute to a more trustworthy IBM result. As many of the key components of IBM workflows have, by now, been relatively widely reported and described, this section will focus on parts of the workflow that are key to the use of historic imagery in such contexts. A summary of the fundamental concepts of the process, particularly as applied to aerial imagery, can be found in Verhoeven *et al.* (2013, 42).

**Fig. 2:**
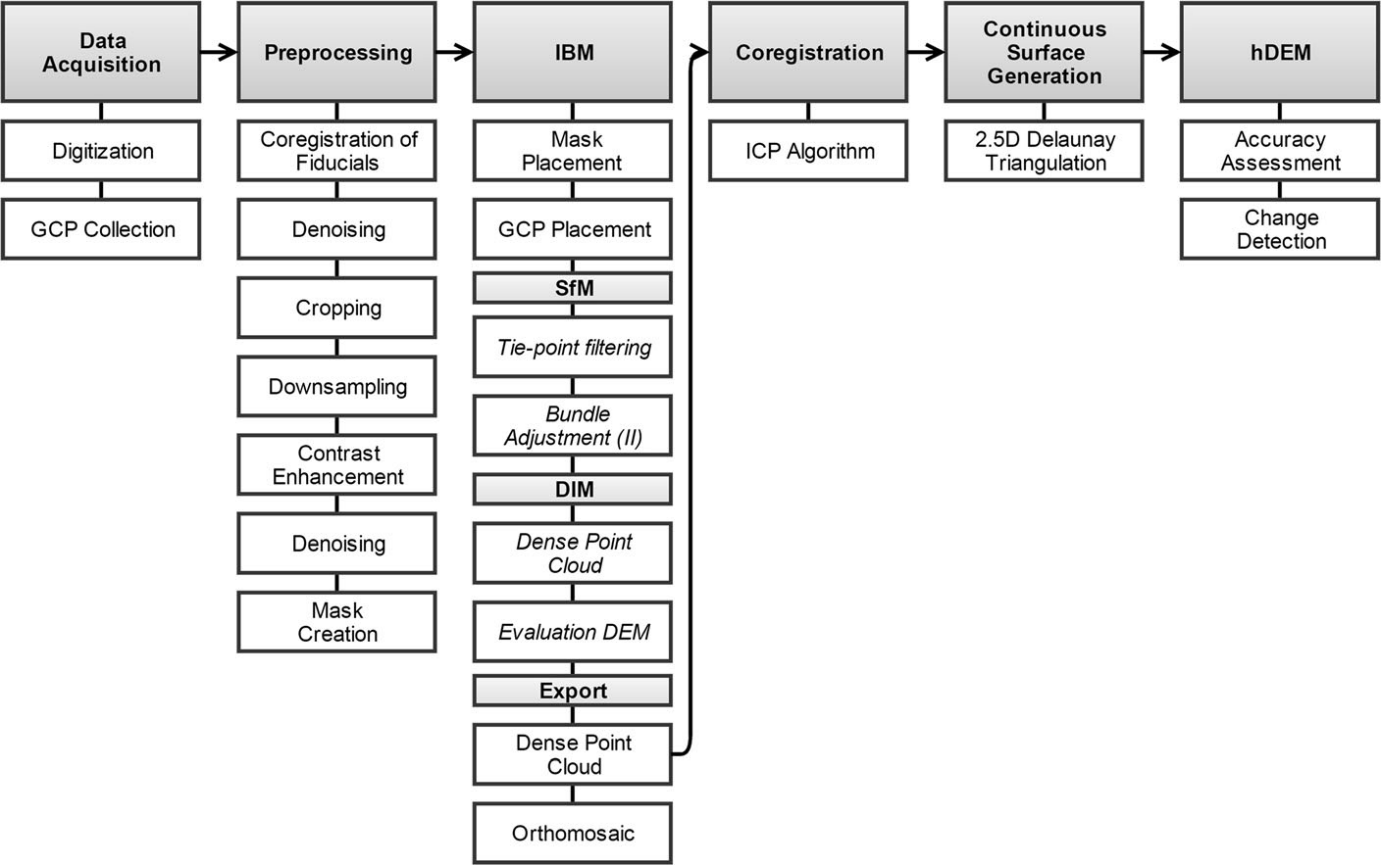
Process for the conversion of historic imagery to georeferenced historic elevation model geocontent. *GCP* ground control point, *IBM* image-based modelling, *SfM* structure from motion, *DIM* dense image matching, *DEM* digital elevation model, *ICP* iterative closest point, *hDEM* historic digital elevation model

For this study, a modified workflow based on Sevara (2013, 405) was implemented. This workflow can be broken down into the following main components: image/data acquisition (discussed above) image preprocessing, image-based modelling, coregistration, and continuous 2.5D surface generation (Fig. 2). Most software used in the implementation of this workflow is either low cost or free/open source, and all software is readily available. Specifics of software packages used for each component of the workflow are given in the subsections below. However, the workflow is designed to be largely software-independent, and there are a number of alternative packages that could be used in each step. It should be noted, however, that due to the large size of the datasets and the processor/memory intensive nature of many of the computational algorithms employed by these software packages, significant processing power may be required in order to obtain high (spatial) resolution results, especially when dealing with large area datasets.

### Image Preprocessing

All historic images were scanned at a resolution of 2500 spi and delivered by the IGM as uncompressed 8-bit .tiff files. As a first step, all images from each flight were coregistered to each other *via* an affine transformation on their fiducial markers and cropped to their original film dimensions. For analogue imagery, this helps IBM software to assume that all images from a series originate from the same camera and have uniform interior orientation parameters (see Sevara 2013, 404 for details). Then, images could be denoised as necessary using a total variation-based denoising algorithm in the software package Fiji, an implementation of the open-source ImageJ image processing platform (Schindelin *et al.* 2012). Next, the original images were downsampled by 50% in order to reduce file size and decrease subsequent processing time specifically for the purpose of terrain generation (note that orthoimage mosaics were created using the original resolution imagery). In addition, this downsampling also effectively removes a good deal of radiometric image noise. In fact, most of the imagery as delivered was, in effect, oversampled (scanned at a higher rate than the original image resolution). After mutually comparing the 3D surface models from both the original and downsampled imagery, no significant reduction in surface detail was found. However, caution should be exercised when downsampling imagery. Original flight height, film and lens resolving power, exposure and many other factors individual to the dataset itself influence the level of detail that can be extracted from a given image set. A balance must always be struck between reduction in as-scanned image resolution and maintenance of feature detail on a per-set basis. Furthermore, as a best practice, analogue imagery should always be first scanned at the native scanner resolution and then downsampled in order to ensure a maximum preservation of detail.

Radiometric optimization of each image was also carried out, in order to increase image contrast and visibility of features, and to counteract vignetting and fading of original image exposure due to the passage of time. The CLAHE (Contrast Limited Adaptive Histogram Equalization) algorithm was used to equalize and improve contrast throughout each image (Zuiderveld 1994). Parameters were derived for each image set through an initial visual estimation and subsequent refinement. Images were processed using the CLAHE implementation in Fiji. A block size of 49 pixels, 256 histogram bins, and a maximum slope of 2.00 were used as processing parameters for each image. After contrast enhancement, further denoising of certain datasets was also carried out in order to further mitigate the effects of scratching and film grain on subsequent feature detection during the IBM process. This proved especially effective for the 1941 image set. All denoising was carried out using an implementation of the total variation-based Rudin-Osher-Fatemi (ROF) denoise algorithm (Rudin *et al.* 1992) in Fiji, using a low theta value to mitigate random noise (such as that produced by film grain) while retaining sharp contrast of real-world feature boundaries.

Finally, an image mask was created for each image set, to be used during the SfM and DIM processes in order to exclude certain areas, such as film borders and areas outside of fiducial markers, from processing. Image masks were created as simple black and white images, with black areas masking out unusable sections of imagery. Use of masks is essential to exclude the use of false tie points along the image borders, *e.g.* at fiducial markers or other elements of the image frame. Mask usage also reduces processing time by reducing the amount of image space the IBM software must consider during feature detection, image matching and 3D reconstruction while simultaneously preserving original image dimensions (Sevara 2013:406; Verhoeven *et al.* 2013:57).

### Image-Based Modelling

After preprocessing, images were loaded into a standard IBM software package for image orientation, and image matching. Agisoft PhotoScan Professional v1.26 was used for the IBM process (Agisoft 2017). Despite a few limitations specific to the processing of historic aerial imagery, notably a lack of the possibility to properly establish interior orientation parameters based on identification of fiducial markers, Agisoft PhotoScan provides a comprehensive package for creation of 3D content from imagery, including SfM and DIM algorithms, ability to place ground control, DEM and orthoimage generation and photogrammetric-style optimization of image orientations (bundle block adjustment). Furthermore, when compared to similar software packages, the algorithms implemented by the various components of the software have repeatedly been shown to be amongst the best performing for both SfM and DIM processes (Remondino *et al.* 2012, 2014).

Images were imported into PhotoScan in separate groups corresponding to their flight dates. After import, image masks were applied to all images in order to constrain the computing cost of feature detection and reconstruction. It is important to note that when importing images of identical dimensions, the software used here will assume that they come from the same camera, even if camera information is unknown. While images from a single vertical mapping or reconnaissance sortie may indeed come from the same camera, localized distortion due to warping or other damage to source media may sometimes necessitate treating these images as if they come from individual (unique) cameras. This allows image interior orientation parameters to be individually calibrated to each image during the SfM step.

After import and masking, GCPs were manually placed in each image so they could be used as constraints in the estimation of accurate image interior and exterior orientations during the subsequent SfM phase. The resulting georeferenced sparse point cloud was manually cleaned to remove outlying tie points with high reprojection error or uncertainty values. The bundle block adjustment was then run again, solving for radial (*k*_1_, *k*_2_, *k*_3_) and decentring (*p*_1_, *p*_2_) lens parameters in order to optimize the image interior and exterior orientations and the corresponding overall root-mean-square error (RMSE) of the model (Table 2). The SfM step was carried out using highest quality settings, while GCP marker accuracy was set to a quality commensurate with the image resolution and quality, in order to avoid model overfitting (Verhoeven and Vermeulen 2016, 751). Next, the DIM was run using the highest possible extraction settings (every pixel reconstructed) and either moderate or aggressive depth filtering.

**Table 2:** Ground control accuracy parameters, RMSE values and average points per square meter after filtering for each dataset

Dataset	Marker accuracy (m)	No. of GCPs	RMSE x (m)	RMSE *y* (m)	RMSE *xy* (m)	RMSE *z* (m)	RMSE 3D (m)	Avg. points/m^2^
22 Apr 1941	0.10	6	0.20	0.07	0.21	0.17	0.27	4
4–13 Jul 1955	0.20	10	0.55	0.76	0.93	0.52	1.07	3
9 May 1975	0.10	22	0.33	0.26	0.42	0.55	0.67	5
24 Jun 1992	0.10	31	0.45	0.50	0.67	0.58	0.89	4

All values in meters unless otherwise noted

Initial hDEMs were calculated directly in the software (as triangular 3D meshes) from the generated dense point clouds in order to visually inspect each dataset for suitability before further processing. Point clouds were then exported in .las format for subsequent coregistration and continuous surface generation in external software. While 3D polymesh surface models or 2.5D raster DEMs can be generated directly in the PhotoScan software, this workflow prefers the coregistration of all point clouds to minimize positional error (see below) prior to 2.5D raster hDEM generation. However, if generating an orthomosaic from historic imagery in PhotoScan, it is necessary to generate a continuous 3D surface model or DEM within the software as a basis for topographic correction of the imagery.

### Coregistration

**Fig. 3:**
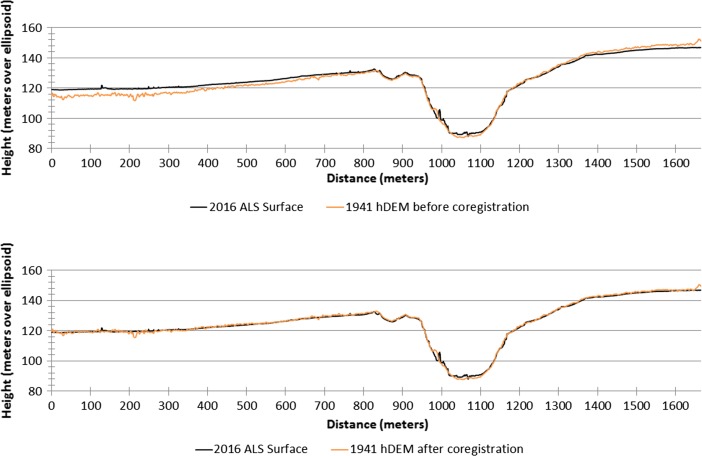
West-east cross section of 2016 and 1941 surfaces before (top) and after (bottom) coregistration. Prior to coregistration, the 1941 surface exhibited a marked tilt despite low control point RMSE values

Once dense 3D point clouds from each historic image set were generated, points from surfaces that appeared to remain constant throughout all periods were checked against corresponding points in the ALS data to estimate correspondence between datasets. A 6-km^2^ area in the centre of our project area, where all hDEM data overlap, was chosen for analysis, and all clouds were clipped to this area. Analysis of distances between historic point cloud data and ALS data showed some residual misregistration between surfaces, despite the high number of GCPs used and the low RMSE values achieved after bundle block optimization. In particular, it can be seen that, although the 1941 dataset had a very low RMSE, the surface was ‘tilted’ in relationship to the ALS reference data, causing unacceptable vertical error in some areas (Fig. 3). This may be due in part to difficulties in accurately locating and placing the ground control in the historic image sets and also to the inability to properly utilize fiducial markers for image orientation within the IBM environment. In order to correct for this, historic point cloud datasets were individually coregistered to a cloud of the last-echo points generated from the ALS dataset using the automated fine registration tool in CloudCompare v2.8, an open-source point cloud and mesh processing software (CloudCompare 2017). This tool uses an iterative closest point (ICP) algorithm with the possibility of adjusting the scale of one dataset to register one point cloud to a reference dataset using a subsample of the point cloud (Girardeau-Montaut 2015:104), which is suitable for clouds where initial coregistration error is minimal. The parameter ‘enable farthest points removal’ was selected in order to prevent the algorithm from attempting to coregister clouds using points sampled from areas of significant landscape change. Application of the tool resulted in a significant reduction in the average distance between clouds. It is important here to minimize distance errors due to misregistration of point clouds that will be used to generate subsequent elevation models while retaining legitimate differences in elevation due to landscape change. Coregistering all datasets to the ALS point cloud also ensures relative accuracy in calculation even in the event that the ALS cloud is not properly georeferenced. In cases where no modern surface data is available, such as when generating all models to be compared using imagery data, one image-based point cloud can simply be used as the reference cloud.

### Surface Generation

Once the coregistration process was completed for each dataset, creation of the 2.5D raster hDEM surface was carried out using the OPALSGrid module of the OPALS software (Mandlburger *et al.* 2009). Individual surfaces corresponding to flight date were interpolated from coregistered point clouds using Delaunay triangulation. All final models were generated at a raster cell size of 50 cm as this was determined to be the acceptable based on the various average filtered point densities per square metre for each cloud and on visual inspection of the data (Table 2). Similar functions can also be carried out in other software packages, including the latest release of CloudCompare (2.8), which includes functions for interpolating surfaces from point clouds (CloudCompare 2017). The resulting surfaces were saved as georeferenced .tiff (geotif) files to be used for analysis in GIS software.

### Results and Data Quality

After elevation model generation, all hDEMs were assessed for quality and accuracy. Positional validation of each model was carried out on each dataset, using the independent checkpoints collected in the field. Additionally, a set of 65 checkpoints referenced to the ALS surface model and randomly placed in open areas of low topographic change were used to estimate vertical deviation of the hDEM surfaces from the ALS surface model. From the RMSE values calculated based on the height position of each surface with regard to either the field or the ALS checkpoint values, it can be seen that there is a marked improvement in the RMSE of most surfaces subsequent to coregistration (Table 3).

**Table 3:** RMSE of surfaces before and after coregistration to ALS reference surface. Note significant improvement in RMSE for all surfaces, with the exception of the 1955 dataset. All values in meters unless otherwise noted. Deviation values are all post-coregistration. *CP* checkpoint, *Min* minimum, *Max* maximum, *Avg* average, *Dev* deviation, *n.a.* not applicable

Dataset	RMSE *z* ALS CP pre-coreg	RMSE *z* field CP post-coreg	RMSE *z* ALS CP post-coreg	Min dev field CP	Max dev field CP	Avg dev field CP	Min dev ALS CP	Max dev ALS CP	Avg dev ALS CP
1941	2.95	0.88	0.67	0.08	1.47	0.44	0.02	1.68	0.5
1955	1.58	1.27	1.35	0.29	2.18	0.64	0.01	3.28	1.06
1975	1.09	0.76	0.49	0.12	1.27	0.54	0.01	1.78	0.36
1992	0.79	0.86	0.41	0.07	1.81	0.34	0.01	1.33	0.31
2016	n.a.	n.a	n.a	0	0.38	0.14	n.a.	n.a.	n.a.

**Fig. 4:**
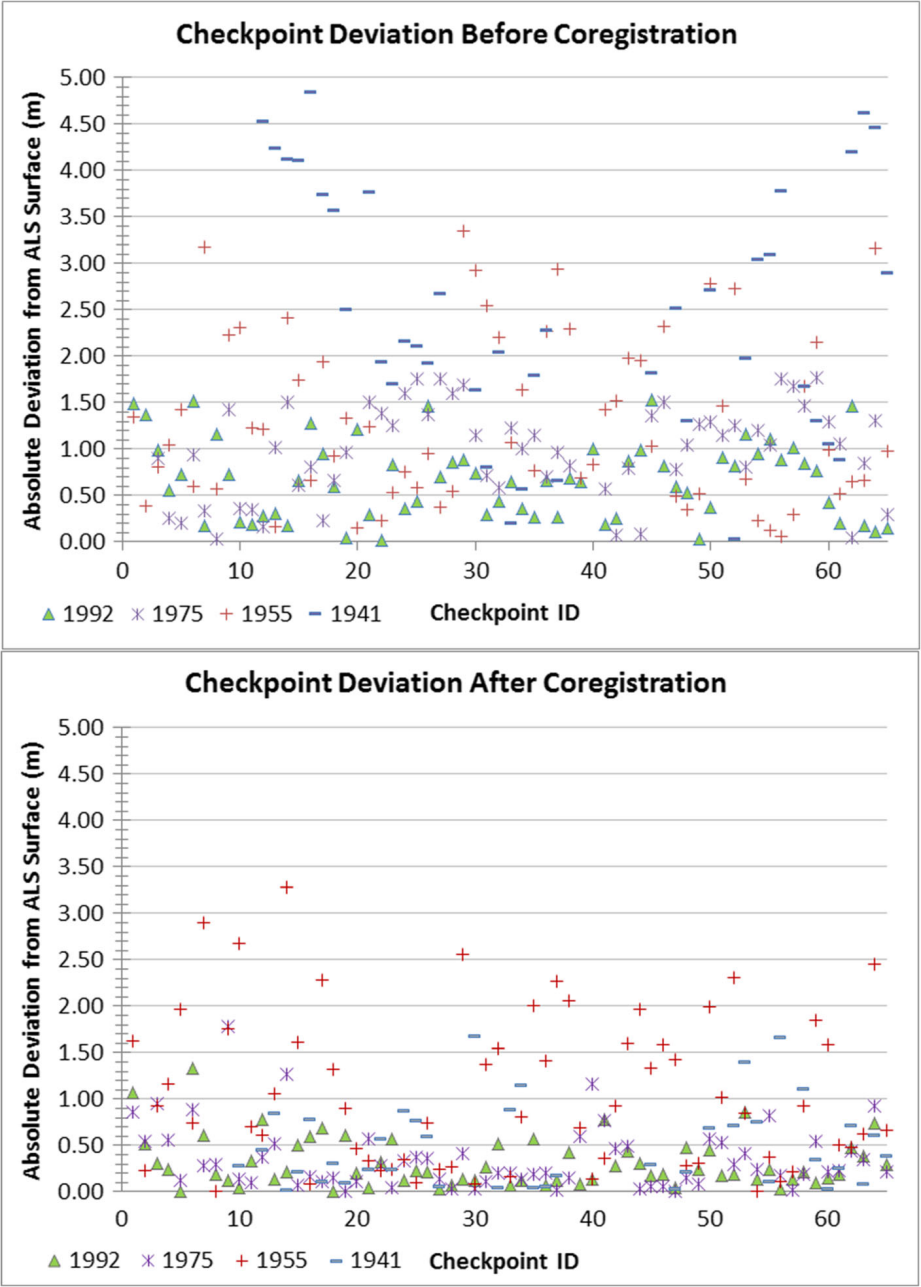
Scatterplot of absolute checkpoint deviations from the ALS reference surface for each hDEM before (left) and after coregistration (right). With the exception of the checkpoints for the 1955 surface, a significant improvement in correspondence between surfaces can be observed after coregistration of the surfaces

In general, we can say that the older image sets used in this study have more performance issues and limitations than younger ones, presumably due to original capture parameters and degradation associated with use and the passage of time. However, it was the dataset from 1955 that performed most poorly in terms of 3D extraction, overall georeferencing RMSE, coregistration and accuracy assessment. Though large topographic features can be clearly distinguished, the overall source image quality is poor and thus produces a noisy surface reconstruction in which features such as buildings are largely not able to be reconstructed. While a marked improvement can be seen in the deviation from the ALS reference surface for most of the checkpoints (Fig. 4), many points in the 1955 dataset show no significant improvement after coregistration. The resulting surface model was thus deemed unreliable and was not used for subsequent analytical purposes. Although the imagery from 1941 originally had extremely high deviation from the reference surface, coregistration of the 1941 surface significantly improved the RMSE. However, the reconstruction quality of the 1941 surface is limited by the overall image quality, which is slightly blurred. This may be the result of a lack of modern forward motion compensation in the equipment used for the original image capture or suboptimal camera settings. The 1975 dataset, flown at a much lower height than the others, provided the most detailed surface reconstruction information; however, the relatively poor radiometric quality, image condition and low side overlap resulted in poor reconstruction in specific areas of the model.

While usable elevation models could be produced from all imagery, it was the 1975 and 1992 imagery that yielded the best models in terms of spatial accuracy. A visual inspection and comparison of image sets and parameters prior to processing could also serve as an indicator of the expectations one can have for the resolution of the resulting data products. For instance, given similar camera focal lengths, as in most of the imagery used in this study, one would not expect the same level of detail to be extracted from imagery captured in 1955 at 6000 m as one would of imagery captured over the same area in 1975 at a flight height of 2500 m, *ceteris paribus*.

This may be particularly apparent with regard to the 1992 imagery used in our study. Although the accuracy assessment indicates that the resulting hDEM has a vertical RMSE of 0.41 m as compared to the ALS surface, this may be beyond the realistic height accuracy limit to be expected from an image set at a scale of 1:36,000, acquired a few decades ago. In the end, the maximum vertical resolution to be expected from an analogue image will be dependent not only upon the final resolving power of the photographic system but also on the contrast of the subject and degradation of the image over time. While the parameters of the photographic system can be modelled with a good deal of accuracy, film degradation over time may impart variables unique to a particular film roll or frame. As a consequence of this, the overall comparability of each dataset to all others is limited by the spatial resolution and the overall accuracy of the lowest quality dataset. Still, this does not preclude datasets emanating from single flights from being used for higher resolution applications.

## Application: Historic Terrain Analysis of the Mazaro River Corridor

### DEM Differencing

**Fig. 5:**
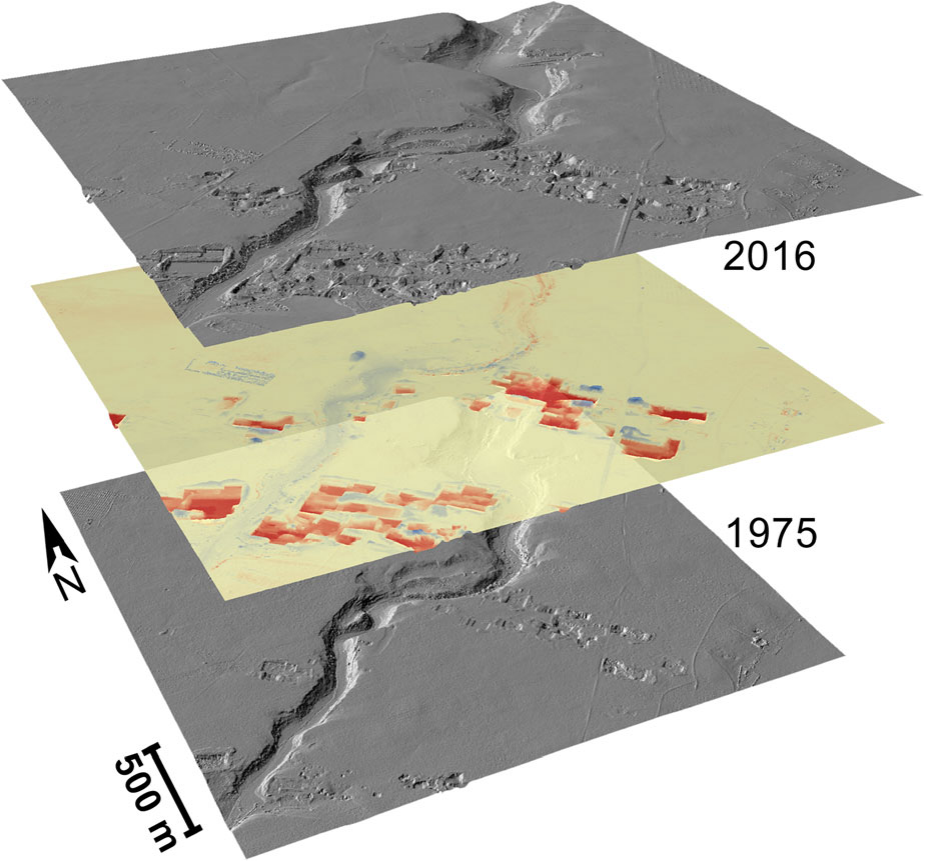
Hillshade representation of a DEM from 2016 (top) and an hDEM from 1975 (bottom), with DEM of difference in middle. Red indicates surface removal (loss); blue indicates deposition (gain). Corresponding surfaces are indicated in yellow

In order to track the physiographic change to the landscape along the Mazaro river corridor from 1941 to 2016, it was first necessary to calculate the changes between each time period as represented by the data in the historic elevation models. In this process, elevation models from each time period are subtracted from their modern counterparts, resulting in a new raster dataset whose values represent the positive or negative per-pixel change between the two models (Fig. 5) (Wheaton *et al.* 2010, 138; James *et al.* 2012:182). The process, conducted by sequentially subtracting an hDEM of a single time period from each of its younger counterparts, resulted in six DEMs of difference, each at a spatial resolution of 50 cm. Here, we use a simple subtraction approach using the raster calculator function in ArcGIS ArcMap 10.4 and tools provided in the GCD software package (GCD 2017); however, this is a mathematical function that can be calculated in any GIS package.

### Geomorphic Change Detection and Impact on the Landscape

**Fig. 6:**
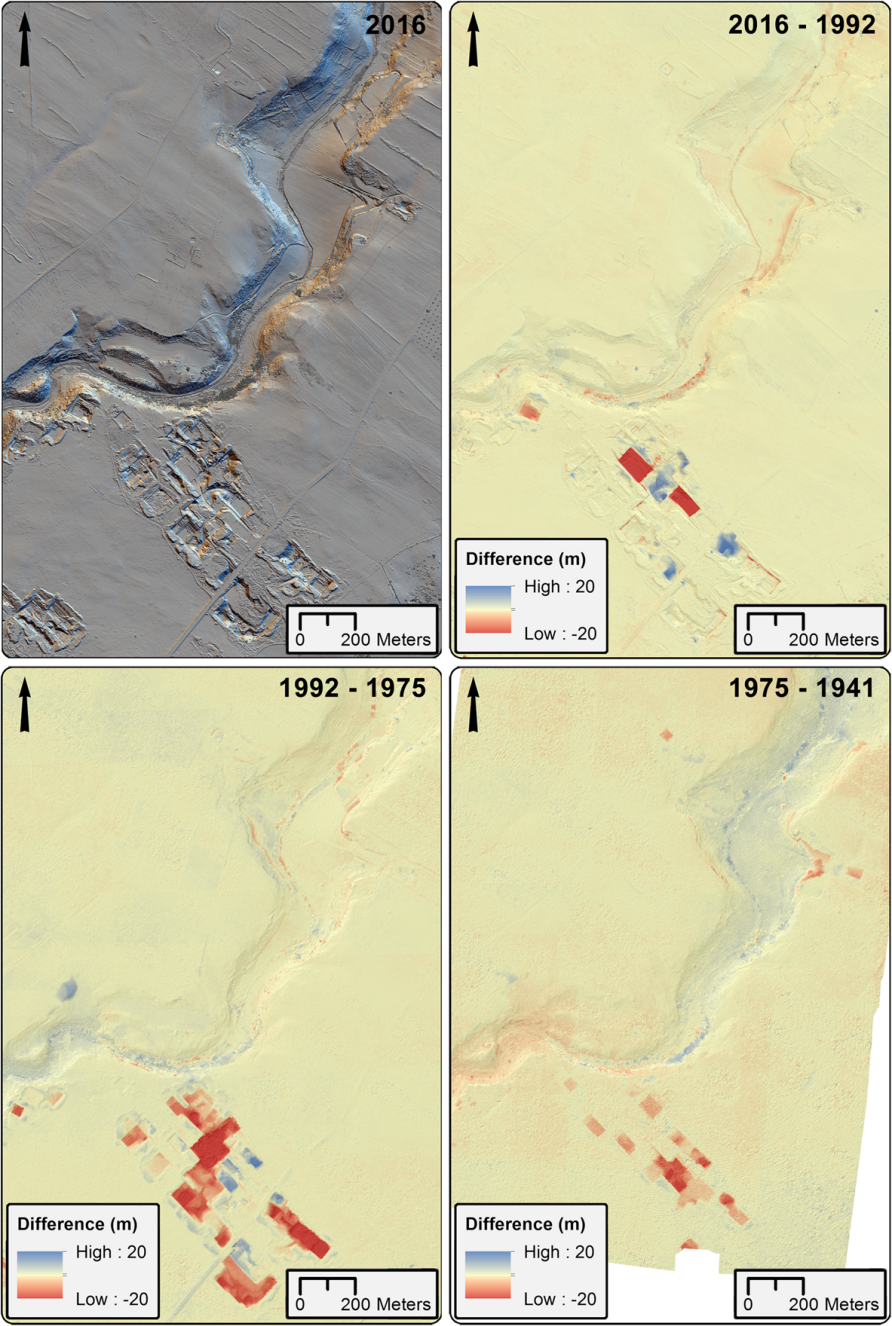
Top left: 16-direction hillshade of the area along the Mazaro river, 2016.Top right: DEM of difference between 2016 and 1992. Bottom left: DEM of difference between 1992 and 1975. Although this is the shortest period investigated, it shows the largest amount of quarrying activities. Bottom right: DEM of difference between 1975 and 1941. The most striking activity is quarrying for calcareous sandstone building material, which is greatly accelerated between 1975 and 1992, and dumping of quarry waste, which has a significant infilling effect from 1992 onward. Areas of accumulation and loss along the Mazaro river are likely due to agricultural activity such as ploughing and backcutting to clear space for planting

DEM differencing represents the first step in the geometric change detection approach we apply, in which we seek not only to quantify the natural and anthropogenically generated differences in the physical landscape over time but also to track their spatial (including volumetric) boundaries (Wheaton *et al.* 2010; Vosselman and Maas 2010, 244). Simply subtracting the oldest dataset from the newest provides an overall calculation of areas of change between two time periods relative to the resolution of the datasets. However, utilizing the whole series of hDEMs provides information about the sequence of landscape change and thus the identification of episodic periods of deposition and removal (Fig. 6), as well as allowing us to estimate periods of time at which landscape change, may have been accelerated. It is even possible to detect areas and periods where removal was followed by infilling, which would be very difficult to recognize solely through observation of the modern landscape. Furthermore, correlating areas of gain or loss between historic datasets can help to identify erroneous areas in individual datasets, where elevation values may be skewed due to processing or source image error. Additionally, using elevation data allows us to estimate the volume of change in addition to its two-dimensional extent, giving us the possibility to consider the depth of change to an area and the likelihood of remaining subsurface archaeological materials.

In order to generate discrete spatial boundaries to help visualize and quantify the topographic change in our study area, each raster difference model was first manually reclassified into 11 classes, using a simple minimum level of detection of 1 m to account for uncertainties created by differing image qualities, geometric resolution, noise from changing vegetation and areas of poor reconstruction between datasets, as well as to account for the much higher level of detail present in the newer imagery and the ALS dataset. This variability means that the lowest quality dataset must be considered as the baseline when comparing multiple sources of input. For this portion of the study, topographic change was classed in 1 m intervals of gain or loss, up to 5 m. Areas of 5 m or greater change were grouped together. Each class represents a gain, loss or no change value. Reclassified raster datasets were then converted to vector data, from which calculations of area per class were computed. At a base level, this resulted in an estimation of per-period percentage of change and relative topographic stability for each period (Table 4). On a more detailed level, both the severity and the spatial extent of topographic change could be mapped in order to help us see where, when and to what extent areas of subsequent human or natural activity may be masking visibility of earlier activity in the landscape.

**Table 4:** Gain, loss and total change of greater than 1 m as measured for six date ranges between 1941 and 2016 in an area of 6 km^2^ along the Mazaro river

Date Range	Total (km^2^)	Area loss (km^2^)	% Loss	Area gain (km^2^)	% Gain	Total % change
1941–1975	3.6	0.81	22.5	0.54	15.0	37.5
1941–1992	3.6	0.65	18.1	0.62	17.2	35.3
1941–2016	3.6	0.56	15.5	0.53	14.7	30.2
1975–1992	6.0	0.72	12.0	0.77	12.8	24.8
1975–2016	6.0	0.69	11.5	0.63	10.5	22.0
1992–2016	6.0	0.36	6.0	0.32	5.3	11.3

This can be further correlated to particular activity types present in our project area, including the predominance of quarrying and the modification of the land surface for cereal growth, olive and evergreen plantation. Areas of extensive soil loss can be seen to mainly be a product of modern quarrying activity, a prominent multiperiod activity in our project area. For instance, a 12% negative change between 1975 and 1992 is largely due to this activity. Loss and gain related to quarrying activity is not linear, with intervals showing modern expansion of historic (and possibly prehistoric) quarries, gain in some areas due to deposition of tailings, and subsequent infilling of quarries that have fallen into disuse (Fig. 6). The hDEM analysis shows that this infilling has accelerated since the 1990s, in conjunction with an apparent slowdown of resource extraction in the area.

**Fig. 7:**
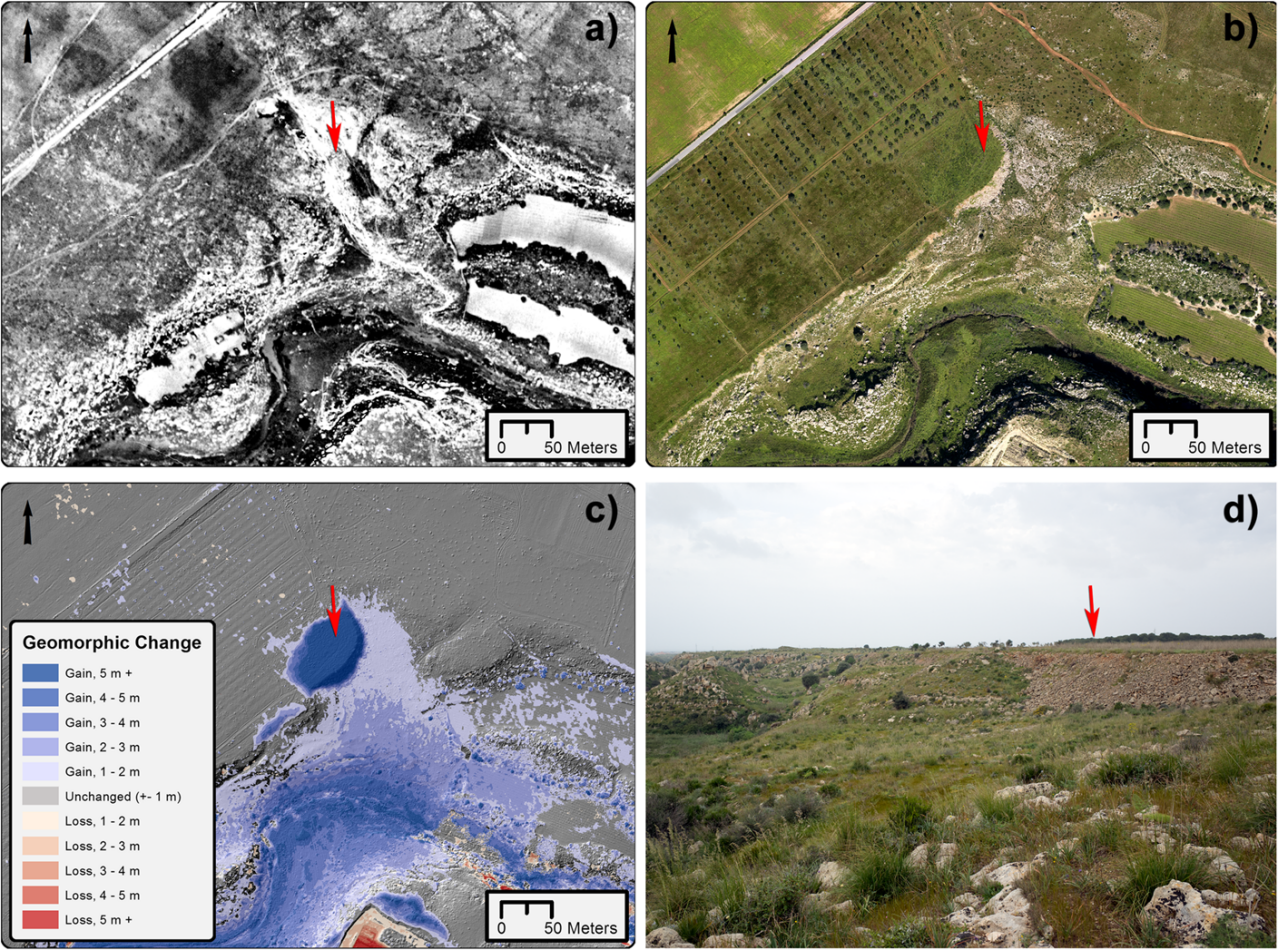
Detail of geomorphic change related to infilling for agricultural purposes. **a** Orthoimage from 1941. **b** Orthoimage from 2016. **c** Geomorphic change. **d** Photograph from April of 2016 showing infilling. Arrows indicate subject depicted in **d**. Image (**a**) source: Istituto Geografico Militare, volo 1941—F.254-serie 4—fot. 107. Used with permission, authorization N. 6933

Significant change can also be seen at discrete areas along the banks of the Mazaro, where mechanical cutting into the banks and cliff face has been carried out in order to increase space for agricultural pursuits closer to the river. Areas of gain are also present, particularly along the edge of the Mazaro plateau, where extensive infilling or flattening of certain areas has preceded plantation of olive or grape vines (Fig. 7), and also in the river valley, where repeated ploughing has begun to produce downslope accumulation of soils. Some gain is also vegetation related, as in areas where tree plantations have grown over the past 60 years or in areas where former vineyards or planting areas have become disused and overgrown.

**Fig. 8:**
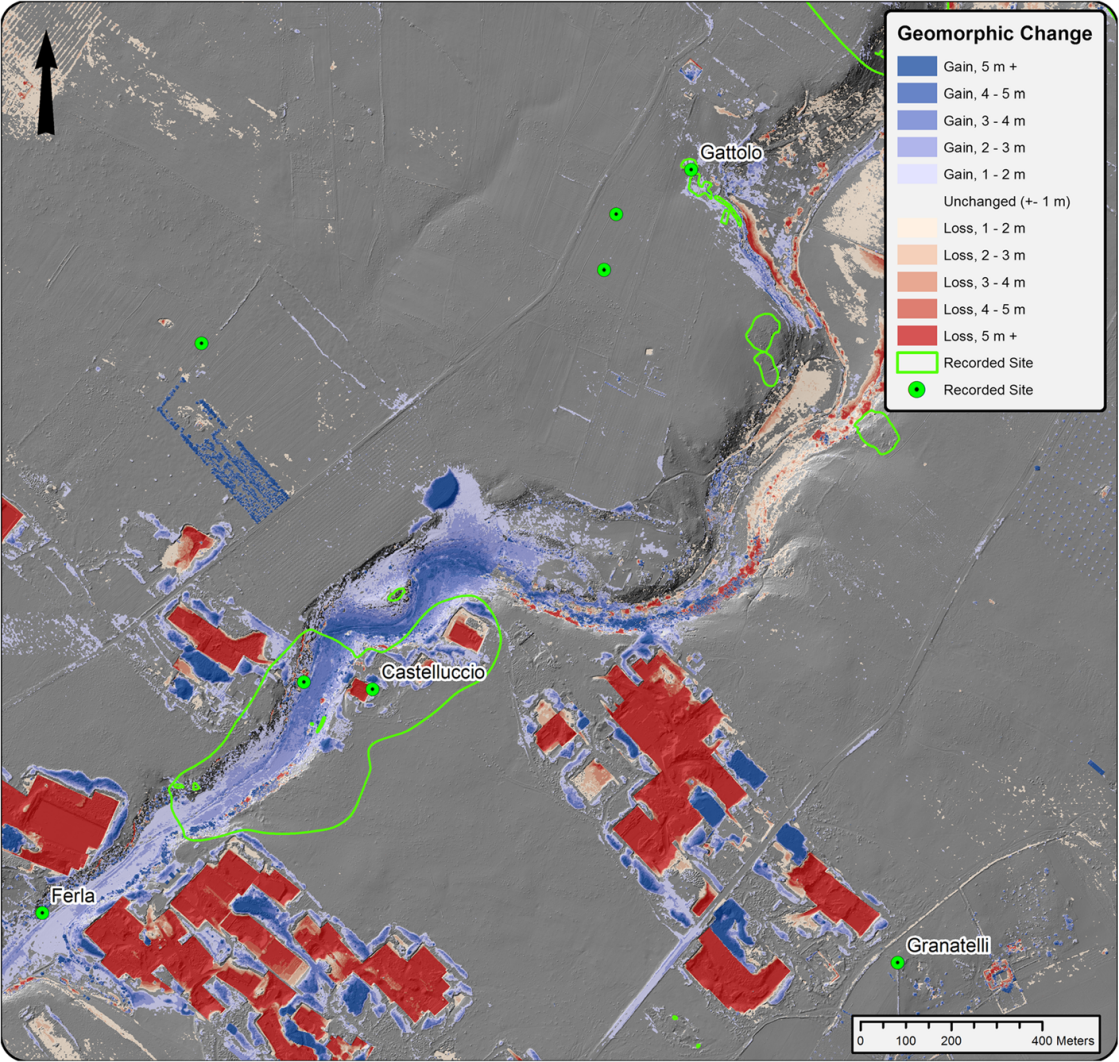
Areas of topographic gain (blue) and loss (red) and known areas of archaeological activity along the Mazaro river between 1975 and 2016, depicted over a 16 direction hillshade of the last echo DSM

In areas of significant change, we can be fairly certain that modern land use has had a great effect upon the preservation or visibility of the bulk of traces of any prior activity in the landscape (Fig. 8). This is particularly apparent in areas with negative change (loss), where postdepositional processes have eradicated many traces of former activity. In some cases, however, although modern quarrying activity may largely remove evidence of prior human activity in the landscape, it can also inadvertently expose it, as is the case in a number of areas where historic and prehistoric features such as aqueducts and tombs are visible in quarry profiles. In areas of significant gain, we may consider the possibility that some archaeological resources may be ‘capped’ by overburden, provided that the deposition was episodic and surface preparation prior to deposition was minimal. Here, we can also consider whether artefacts found on the surface may have any correlation to subsurface remains. Therefore, we may consider areas of significant topographic change less in relationship to a solely presupposed binary presence/absence of prior activity indicators, but rather that traces of past human activity that remain in these areas may manifest in remarkably different and more limited ways than in other parts of the study area. However, although traces may not be altogether obliterated, there can be little doubt that severe geomorphic changes such as those brought about by extensive quarrying have had a significant effect on preservation of traces of preceding human activity. While we cannot know this directly through observation of the modern landscape, locations of recorded archaeological sites that have subsequently been affected by modern landscape change can be used in conjunction with historic elevation models to examine such change at more local levels.

### Local Effect on Archaeological Resources: the Site of Gattolo

hDEM data can also be used on a more detailed level to examine topographic change and its impact at individual archaeological sites in the project area. Given the original scale and condition of much of the imagery used in our study, the average point densities and the resolution of the resulting elevation models, we cannot expect high-resolution feature extraction for monitoring at the centimeter or decimetre level over the entire project area. Nevertheless, in discrete areas, we can use some of these models to track topographic change at a more detailed level. One example of this is the Bronze Age site of Gattolo (Calafato *et al.* 2001). Gattolo is characteristic of known archaeological resources in the region, as it contains human activity form a long period of time, has mortuary components built in to the cliff face along the river and is a nucleation point in the landscape whose later periods of occupation mask earlier activity (Fig. 8).

The site of Gattolo sits on the west bank of the Mazaro, at the edge of the river. At Gattolo, existing published information indicates a number of prehistoric rock cut tombs, all of which are currently in states of partial or near total destruction due to subsequent agricultural activity and other disturbance. The area also has an extensive historic component, as evidenced by a number of buildings that appear on historic maps from the 1800s. A large rural farmhouse, now abandoned, sits just above the location of the tombs. The area around Gattolo has been extensively modified for agricultural and habitation purposes since the 1950s, and historic aerial imagery allows for the reconstruction of the sequence of landscape modifications. The large rural farmhouse does not appear on the earliest imagery of the area; however, it is present by 1955. Extensive modification of the terrain around the farmhouse ensued between 1955 and 1992, when the area to the northwest was flattened for olive plantation, and overburden was brought in to level out the plantation area from behind the house to the edge of the Mazaro.

**Fig. 9:**
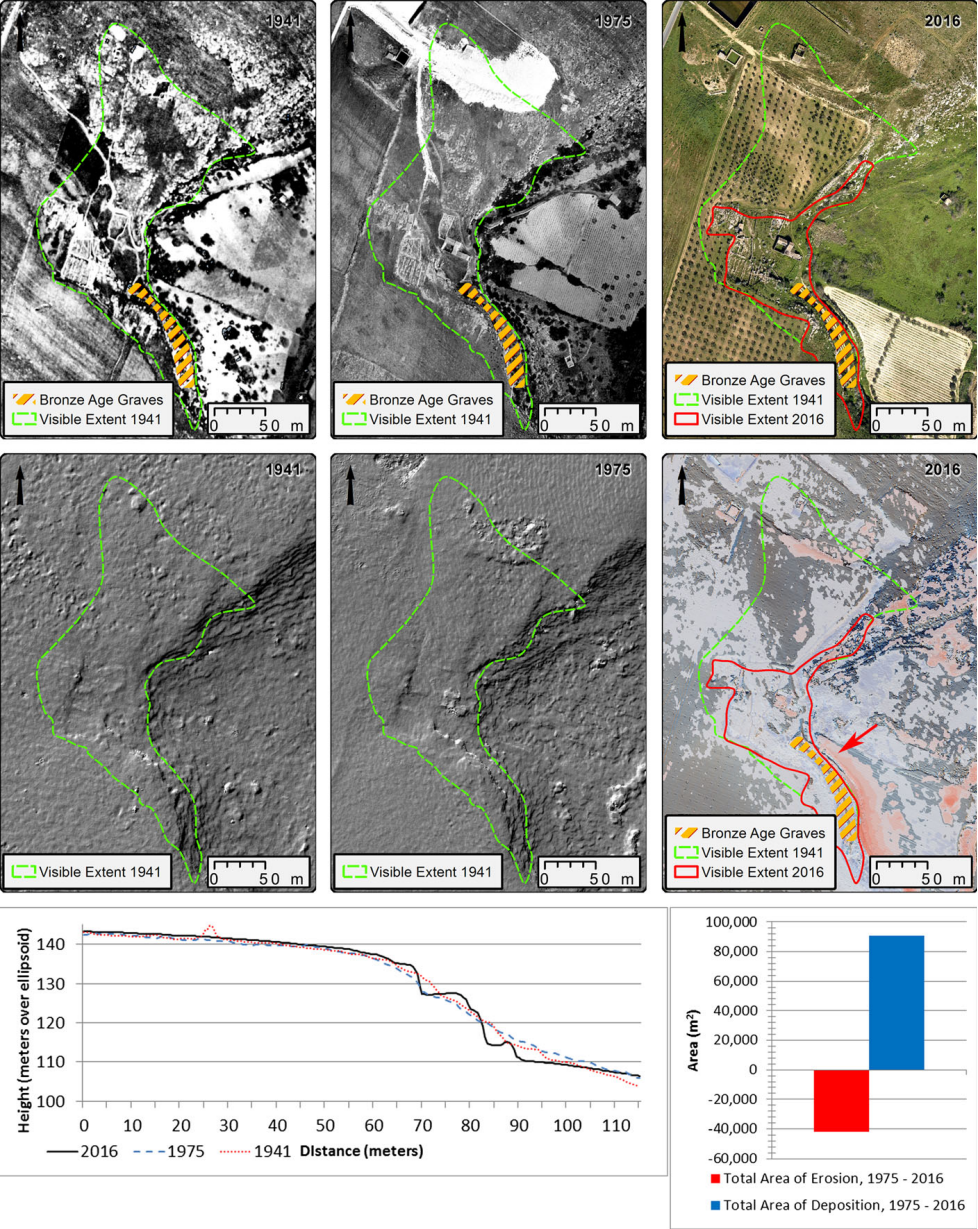
Top row: Visible extent of Gattolo in 1941 (left), 1975 (centre) and 2016 (right). Middle row: Hillshade of hDEM showing surface at Gattolo in 1941 (left) and 1975 (centre). Right image shows an ALS derived hillshade of Gattolo in 2016, showing areas of deposition (blue) and erosion (red) between 1975 and 2016 using a minimum level of detection of 50 cm. Darker colours indicate higher gain/loss, see Fig. 8 legend for reference. Bottom row: Profile view of Bronze Age tomb area between 1941 and 2016 (left) and chart showing the total area of erosion and deposition in the vicinity of Gattolo between 1975 and 2016 (right). Red arrow in centre right image shows profile location. Top left, top centre image source: Istituto Geografico Militare, volo 1941—F.254-serie 4—fot. 107, volo 1975—F.257—serie VIII—fot. 771. Used with permission, authorization N. 6933

The area around the Bronze Age tombs was heavily cut back at some point in the middle of the last century to create a flat terrace for viticulture, exposing and partially destroying the tombs and their original contexts (Ingoglia and Tusa 2006). Partial excavation of one exposed tomb in 1985 resulted in the recovery of *in situ* ceramic material dating to the Early Bronze Age (Calafato *et al.* 2001, 38; Ingoglia and Tusa 2006; Tusa 1999, 442). While the Bronze Age tombs below the house were previously known, project fieldwork in 2016 documented extensive evidence of previously unrecorded exposed settlement structures of unknown period, in the form of rock cut foundations, in the numerous locations around the farmhouse. Visual inspection of the historic imagery shows similar features to have been more extensively visible prior to modification of the land for agricultural purposes, indicating that the site area may have been much more extensive and exposed than it is today (Fig. 9, top left). Profile reconstructions based on hDEM data also allow us to track removal of material in the area of the Bronze Age tombs (see profile graph; Fig. 9).

The change detection analysis shows corresponding overburden of up to ca. 1.3 m over the formerly exposed possible site area since 1975 alone, allowing us to infer that parts of the site may have been capped and thus possibly protected by the accumulated material (Fig. 9). Due to the higher level of detail present in the 1975 imagery, here we are able to use a minimum level of detection threshold of 50 cm. Corresponding in-field GNSS measurements indicate that the overburden in this area to be between 0.9 and 1.5 m as measured along the edge of the upper bank. Overall, calculations show that ca. 52% the site area has undergone some form of topographic change for the same period. While the level of deposition seems to correspond with fairly uniform distribution of material over the site in preparation for olive planting and vegetation growth, the erosion seems to mainly be a product of the aforementioned extreme backcutting near the river. Thus, the historic elevation data used in this study is able to provide us with information in two key ways: Not only does it provide us with a calculation of soil loss and deposition in the vicinity of the site, it also provides us with a historic topographic surface that can allow us to reconstruct the previous extent of the site.

## Discussion

### hDEMs: Merits and Limits of the Approach

Although the merits of generating historic elevation models for landscape analysis are numerous, there are still a number of limitations that need to be addressed. Some of these are limitations of the computational methods used in this study and as such can be addressed and potentially mitigated in future work. Others are limitations of the source material, which should be understood when considering scales and possibilities of recovery of 3D data from historic imagery in general, and are thus largely method independent.

To begin with, the methods used in this study, while highly automated in part, are still comparatively time consuming. Due to the nonuniform nature of the preservation quality of most historic imagery, all images must be individually examined and parameters for image preprocessing usually have to be tested in an iterative manner before optimal parameters can be found. This includes settings for contrast enhancement, downsampling and denoising as well as evaluating their effects on the final hDEM products. Since the mandatory photogrammetric scanning at small sampling intervals generates very large images, these tasks can be financially and computationally expensive.

There are also a number of technical issues relating to the IBM process that should be addressed in order to improve the overall process and results of modelling terrain from historic imagery. Firstly, the approach used in this study still does not fully utilize fiducial markers present in imagery. The ability to do this could vastly improve interior orientation estimates and camera positioning. This, in turn, could lead to more accurate hDEM surface reconstruction. A second issue is that of ground control GCP placement. In the workflow presented here, ground control is still placed manually, a time consuming and sometimes error-prone process. In some studies, in-field acquisition of ground points may not even be feasible. OrientAL, an open-source software for automated orthorectification of historic and modern vertical and oblique aerial imagery that uses SfM algorithms for image orientation, is one potential solution to these issues (Karel *et al.* 2013, 2014). OrientAL can automatically identify fiducial markers in scanned historic aerial imagery and use these as constraints in calculation of interior orientation. Additionally, OrientAL contains routines for semi-automated georeferencing of imagery using a modern DEM and the selection of just a few reference points. Currently, OrientAL is designed to function with images from the Aerial Archive at the University of Vienna; however, its functionality will be expanded in the future to include other sources of historic imagery. These issues, along with more in-depth examination of the effects of image preprocessing on archival media, will be taken up in a subsequent study.

Another issue is that of data dimensionality. In this study, when we create hDEMs, we only work with 2.5D data, and we effectively discard one half of a dimension. The main benefit of this is one of time and processing power, as generating a full high-resolution 3D mesh of a large area and subsequently analyzing it would be computationally cumbersome. However, this means that objects with complex, overhanging geometry are not properly modelled (Verhoeven 2016). As the current study exclusively employs vertical or near-vertical imagery taken from a comparatively high altitude with fairly moderate overlap as a basis for feature reconstruction, a full 3D surface would likely yield little added data value for the increased complexity of the application. Results of a fully 3D approach might see far more merit when applied to data derived from oblique imagery or highly overlapping combinations of oblique/vertical images.

The parameters of original image capture are also critical to the overall model outcome. Image scale, flight height, resolving power of the imaging system, overlap and other factors noted above all play an important role in the subsequent quality and geometric resolution of historic terrain models. Other factors, such as time of year of image capture, also play an important role in the potential manifestation of indicators of subsurface archaeological activity. When capturing imagery specifically for archaeological purposes, we can attempt to take many of these factors into account and to compensate for them. The materials used in our study were not flown for archaeological purposes and thus may not have been collected at optimal times of year for certain factors such as vegetation and soil mark manifestation; nevertheless, all were captured specifically for mapping purposes by the IGM and thus were originally well exposed and flown at a time of day with minimal shadowing. However, this will not always be the case with historic imagery, particularly archival wartime reconnaissance images. All of these factors should be considered when assessing the fitness for purpose of such an approach for any given image set.

While the relatively poor geometric quality of older historic aerial imagery has been cited as a reason for the adequacy of high-end nonphotogrammetric scanners for softcopy photogrammetric applications (Nocerino *et al.* 2012, Redecker 2008), from an archival standpoint, it would seem to be most sensible to attempt to preserve copies of analogue imagery in digital form with as little induced distortion as possible. Given the factors of degradation discussed above, scanning at the highest possible geometric quality would best preserve imagery for future use, even if such quality exceeds the immediate needs of a given project. It could also limit the amount of subsequent scanning that might need to be carried out on analogue materials. Nevertheless, in certain circumstances, the option to use photogrammetric-grade digitization platforms may not be available, and in these cases, a workflow that uses some form of image or hardware calibration for nonphotogrammetric scanning (*e.g.* Mitrovic *et al.* 2004; Nocerino *et al.* 2012, 87) could also improve or at least minimize the effect of such geometric distortions on digitized imagery. However, scanning of imagery using photogrammetric grade devices should be considered as an overall best practice when at all possible. Moreover, it is important to remember that even the highest quality digitization platforms cannot improve or eradicate geometric distortion already present in the source media and that the platforms themselves will actually introduce minute amounts of new geometric deformation.

Finally, it should be noted that only what the image captures can be reconstructed. Even under ideal circumstances, the best quality imagery will never provide the full picture. Although a valuable source of information, the outcomes of any reconstruction based on historic imagery should always be evaluated with these factors in mind. In short, we should ask ourselves: What do we gain from observing the process of landscape change *via* historical elevation models that we cannot understand from simply viewing its result and can the available material provide that information?

### The Future of the Visual Past: Historic Terrain as Source Criticism

Even given the constraints discussed above, the overall geomorphic change maps and the site level change are helping us to understand how those changes have had an impact on what evidence may remain of prior activity in the landscape. Along the Mazaro, the two main processes of extensive surface modification are quarrying and, in the last decades, increasing agricultural surface alteration. We can see that modern activities such as quarrying take place in largely the same areas as their historic counterparts due to the continuing availability of suitable sandstone. From what we see in the available air photos, the physical process of landscape change was at its greatest between the 1975 and the 1992. This change is, of course, only one of many processes, both physical and perceptual, which affect how we see and interpret the modern distribution of the present remains of past activity in the landscape (Cowley 2016, 59; Meinig 1979). However, in our project area, this change has had a significant physical effect on the environment, and identification of these processes and their impacts will greatly assist the subsequent assessment of the effects of this change on our documentation and interpretation of the archaeological record. The geomorphic change calculations obviously show large-scale and large volume modifications (*e.g.* Figs. 6 and 8). Although it is not explicitly discussed here, these models can also help us to track subtle erosion and deposition occurring as a result of nonhuman environmental processes in the landscape, a topic which will be explored in greater detail as our work in the project area progresses.

These datasets are therefore very useful for helping us to understand the current spatial distribution of remaining archaeological resources in our project area. In areas where the surface has been capped by significant deposits, for example, historic DEM data could be used to estimate the degree of likelihood that any material found therein is related to its original spatial context. Furthermore, depending on the disturbance to the original ground surface, we can consider the possibility that buried archaeological remains may be accessible in these areas *via* other means, such as excavation or geophysical prospection. Conversely, areas that exhibit extensive signs of recent extraction of surface material, such as the modern quarry areas discussed above, can be shown to have a lower likelihood of meaningful surviving prior archaeological remains within their bounds. This does not mean that these should be disregarded out of hand; rather, in conjunction with other information, hDEM data can help us to infer why we may not see certain periods of activity in parts of the landscape. The discussion of the value of objects such as these as relicts of industrial heritage in and of themselves is, of course, another thoroughly valid line of inquiry, for which hDEMs can be used to track their historical development. Additionally, historic elevation models have the possibility to provide topographic information about previous iterations of the landscape that can be used as elements in regression/retrogressive analysis of historic landscape features, data that can in turn be useful for broad brush applications such as historic landscape characterization.

These models are important from a continuity standpoint as well, helping us to build a picture of the areas that may not have been significantly affected by modern land use processes and where the ground surface may be more contiguous with earlier periods. Overall, we can see stability and change in different parts of our project area as relating to different natural properties and the changing economic and cultural value of the land. Of course, the bias inherent in the imagery itself must also be taken into account when assessing the information gained from these sources. This is why we prefer to think of this material as helping us to mitigate effects of land use bias rather than eliminate them.

In 2002, van Leusen noted that the influence of ‘subsequent human occupation and land use’ on prior remains in the landscape was ‘severely understudied’ in the Mediterranean archaeological tradition (van Leusen 2002, chapter 4, page 17). Through the creation of elevation models from historic map data, his study was able to show the effectiveness of tracking landscape change caused by modern land use activities as a way to understand how recent physical change both hid and redistributed archaeological resources (van Leusen 2002, chapter 11, page 17). Here, we have shown that this can also be carried out using elevation models generated from historical imagery and that a sequence of such data gives us insight into the nonlinear nature of landscape formation in our project area. A sequence of hDEMs can therefore act as a form of source criticism for a given landscape, with each discrete period of activity documented through imagery acting as an independently created eyewitness event (with some known parameters and limitations) that can be compared to its preceding and subsequent counterparts and juxtaposed with other data sources. Thus, this allows us to evaluate the processes that contributed to the shape of the landscape as we see it today from multiple temporal perspectives and compare these with what we see in the present. This gives us the ability to at least partially understand and deconstruct the *process* of modern landscape formation and its impact on archaeological resources, rather than simply viewing the present as a *result* of past activity. In this way, reconstructions of the physical changes to the landscape act as narrative points in its biographical sequence. Furthermore, we can use this sequence as one of many ways to understand and at least partially mitigate the effect that subsequent human land use may have on our understanding of preceding episodes of human activity.

## Conclusion

We have presented a workflow for the accurate reconstruction of historic terrain from vertical aerial photographs for the purposes of landscape analysis using a readily available computer vision-based process and preprocessing techniques designed to specifically address a number of the unique needs of historic analogue aerial imagery. Using this process, we have shown that computer vision-based applications can be a viable solution for the purpose of generating elevation models for historical terrain analysis. Moreover, the workflow applied here utilizes low-cost and readily available software packages to accomplish these tasks, providing an accessible, repeatable and accurate way to recover detailed, quantifiable information about physical parameters of past environments from historic imagery. Therefore, we find that our approach to hDEM reconstruction from different generations of historic aerial photography and the calculation of DEM differencing from these time slices is an efficient and straightforward method for the differentiation between (i) more or less intact surfaces, (ii) those which have been covered and (iii) areas where surfaces have been completely destroyed.

The applicability of this approach has been demonstrated at multiple scales using a series of historic images covering our research area in western Sicily, where a temporal sequence of five elevation models generated from data captured between 1941 and 2016 has helped us to identify the extent and depth of physical changes to the landscape in our project area and to evaluate the effect of these processes on specific archaeological sites in addition to the wider landscape. From this, we have been able to quantify the extent of topographic loss and gain due to anthropogenic activities such as resource extraction and changes in agricultural practice and to show how the nonlinear nature of these events affects our perception of the present remains of past activity in the intermediate zone between the coast and the interior. Furthermore, as this differentiation allows for the calculation for the extent and depth of extraction/deposition, it can provide fundamental data for any scientific field concerned with changes of the Earth’s surface, including geomorphology (for applications such as documentation of gravity induced deformation, glacier flow, fluvial processes, dune movements, coastal retreat), environmental studies, land development and cultural heritage management.

In addition to representing physical changes to the environment at discrete points in time, information gained from this research has helped us to identify the extent to which certain postdepositional processes may influence the recovery and visibility of earlier activity in the landscape. Thus, this approach may be particularly suitable for historic landscape analysis in areas that have undergone significant and rapid morphological change within the last century, at the confluence of agricultural intensification, rapid urbanization, human population growth and the advent of systematic visual coverage of the surface of the Earth. This also means that the physical parameters of many of our vanished historic landscapes may not be irrevocably lost, but merely waiting to be translated from mute witnesses of events long past into active informants in our search to understand environments lost to the subsequent activities of people, natural processes and the passage of time.
